# Supplementary material for: Effect of sustained virologic response on liver-related mortality among individuals living with hepatitis C by treatment era: A population-based retrospective cohort study
Source: PLoS One. 2025 Oct 6;20(10):e0333584. doi: 10.1371/journal.pone.0333584 (PMC12500089; doi:10.1371/journal.pone.0333584)
Supplement: S1 Table — (PDF) [file pone.0333584.s001.pdf]

Table S1. Drug identification numbers used to identify HCV antiviral treatment

DIN	DRUG TYPE	GENERIC NAME (BRAND NAME)
**DAA-BASED TREATMENTS**		
**2370816**	DAA (First generation)	BOCEPREVIR (VICTRELIS)
**2371448**	DAA (First generation)	BOCEPREVIR/PEG-INTERFERON ALFA-2B/RIBAVIRIN (VICTRELIS TRIPLE)
**2371456**	DAA (First generation)	BOCEPREVIR/PEG-INTERFERON ALFA-2B/RIBAVIRIN (VICTRELIS TRIPLE)
**2371464**	DAA (First generation)	BOCEPREVIR/PEG-INTERFERON ALFA-2B/RIBAVIRIN (VICTRELIS TRIPLE)
**2371472**	DAA (First generation)	BOCEPREVIR/PEG-INTERFERON ALFA-2B/RIBAVIRIN (VICTRELIS TRIPLE)
**2371553**	DAA (First generation)	TELAPRAVIR (INCIVEK)
**2416441**	DAA	SIMEPREVIR (GALEXOS)
**2418355**	DAA	SOFOSBUVIR (SOVALDI)
**2456370**	DAA	SOFOSBUVIR/VELPATASVIR (EPCLUSA)
**2467542**	DAA	SOFOSBUVIR/VELPATASVIR/VOXILAPREVIR (VOSEVI)
**2432226**	DAA	LEDISPAVIR/SOFOSBUVIR (HARVONI)
**2436027**	DAA	DASABUVIR/OMBITASVIR/PARITAPREVIR/RITONAVIR (HOLKIRA PAK)
**2447711**	DAA	OMBITASVIR/PARITAPREVIR/RITONAVIR (TECHNIVIE)
**2444747**	DAA	DACLATASVIR (DAKLINZA)
**2444755**	DAA	DACLATASVIR (DAKLINZA)
**2452294**	DAA	ASUNAPREVIR (SUNVEPRA)
**2451131**	DAA	ELBASVIR/GRAZOPREVIR (ZEPATIER)
**2467550**	DAA	GLECAPREVIR/PIBRENTASVIR (MAVIRET)
**RIBAVIRIN**		
**2425890**	RBV	RIBAVIRIN (IBAVYR)
**2425904**	RBV	RIBAVIRIN (IBAVYR)
**2439212**	RBV	RIBAVIRIN (IBAVYR)
**INTERFERON-BASED TREATMENTS**		
**2242966**	PEG-IFN	PEG-INTERFERON ALFA-2B (UNITRON PEG)
**2242967**	PEG-IFN	PEG-INTERFERON ALFA-2B (UNITRON PEG)
**2242968**	PEG-IFN	PEG-INTERFERON ALFA-2B (UNITRON PEG)
**2242969**	PEG-IFN	PEG-INTERFERON ALFA-2B (UNITRON PEG)
**2248077**	PEG-IFN	PEG-INTERFERON ALFA-2A (PEGASYS)
**2248078**	PEG-IFN	PEG-INTERFERON ALFA-2A (PEGASYS)
**2239730**	IFN/RBV	INTERFERON ALFA-2B/RIBAVIRIN (REBETRON)
**2241159**	IFN/RBV	INTERFERON ALFA-2B/RIBAVIRIN (REBETRON)
**2253410**	PEG-IFN/RBV	PEG-INTERFERON ALFA-2A/RIBAVIRIN (PEGASYS RBV)
**2253429**	PEG-IFN/RBV	PEG-INTERFERON ALFA-2A/RIBAVIRIN (PEGASYS RBV)
**2254573**	PEG-IFN/RBV	PEG-INTERFERON ALFA-2B/RIBAVIRIN (PEGETRON REDIPEN)
**2246026**	PEG-IFN/RBV	PEG-INTERFERON ALFA-2B/RIBAVIRIN (PEGETRON)
**2246027**	PEG-IFN/RBV	PEG-INTERFERON ALFA-2B/RIBAVIRIN (PEGETRON)
**2246028**	PEG-IFN/RBV	PEG-INTERFERON ALFA-2B/RIBAVIRIN (PEGETRON)
**2246029**	PEG-IFN/RBV	PEG-INTERFERON ALFA-2B/RIBAVIRIN (PEGETRON)
**2246030**	PEG-IFN/RBV	PEG-INTERFERON ALFA-2B/RIBAVIRIN (PEGETRON)
**2254581**	PEG-IFN/RBV	PEG-INTERFERON ALFA-2B/RIBAVIRIN (PEGETRON)
**2254603**	PEG-IFN/RBV	PEG-INTERFERON ALFA-2B/RIBAVIRIN (PEGETRON)
**2254638**	PEG-IFN/RBV	PEG-INTERFERON ALFA-2B/RIBAVIRIN (PEGETRON)
**2254646**	PEG-IFN/RBV	PEG-INTERFERON ALFA-2B/RIBAVIRIN (PEGETRON)
**2223406**	IFN	INTERFERON ALFA-2B (INTRON-A)
**2238674**	IFN	INTERFERON ALFA-2B (INTRON-A)
**2238675**	IFN	INTERFERON ALFA-2B (INTRON-A)
**2240693**	IFN	INTERFERON ALFA-2B (INTRON-A)
**2240694**	IFN	INTERFERON ALFA-2B (INTRON-A)
**2240695**	IFN	INTERFERON ALFA-2B (INTRON-A)

Abbreviations: DIN: Drug identification number; DAA: direct-acting antivirals; PEG-IFN: pegylated interferon; IFN: Interferon; RBV: ribavirin.
