# Supplementary material for: Effect of sustained virologic response on liver-related mortality among individuals living with hepatitis C by treatment era: A population-based retrospective cohort study
Source: PLoS One. 2025 Oct 6;20(10):e0333584. doi: 10.1371/journal.pone.0333584 (PMC12500089; doi:10.1371/journal.pone.0333584)
Supplement: S2 Table — (PDF) [file pone.0333584.s002.pdf]

Table S2. Diagnostic and procedure codes used to identify HCV-related diagnosis and comorbidities from datasets held at ICES

Condition	Dataset	ICD-9	ICD-10	Procedure Code	Intervention Code	OHIP Fee Code	OCR	Death Registry Code for Liver-related death
**Cirrhosis**	**DAD<br>SDS<br>NACRS**	4561<br>5712<br>5715	I859<br>I982<br>K703<br>K746<br>K717	-	-	-	-	-
	**OHIP**	-	-	-	-	571	-	-
**Decompensated Cirrhosis**	**DAD<br>SDS<br>NACRS**	4560<br>4562<br>5722<br>5723<br>5724<br>7824<br>7895	I850<br>I9820<br>I983<br>I864<br>K766<br>K767<br>K721<br>K729<br>R17<br>R18	1006<br>6691	1NA13BAFA<br>1NA13BAX7<br>1NA13BABD<br>1KQ76GPNR<br>1OT52HA	-	-	-
	**OHIP**	-	-	-	-	J057<br>Z591<br>571*	-	-
	**ORDG**	-	-	-	-	-	-	**Main cause of death**: 5715, 5712, 5722, 5723, 5724, 5728, 4560<br>**Other cause of Death:** K721, K729, K703, K704, K717, K74, K746, K766, K767, I85X, I982X, I983, I864
**Hepatocellular carcinoma**	**DAD<br>SDS<br>NACRS**	155	C229<br>C220<br>81703<br>81803	-	-	-	-	-
	**ORGD**	-	-	-	-	-	-	**Main cause of death:** 1550<br>**Other cause of Death:** 81703, 81803
	**OCR**	-	-	-	-	-	**Morphology:** 81703, 81723, 81733 81743, 81753, 81803<br>**Topology:** C220	**Morphology:** 81803 or 81703,
**Liver transplant**	**DAD<br>SDS<br>NACRS**	V427<br>99682	Z944<br>T8640<br>T8641<br>T8642<br>T8643<br>T8649<br>T869	624x	10A85LAXXK<br>10A85VCXXK<br>10A85WLXXI<br>10A85WLXXK	-	-	-
	**OHIP**	-	-	-	-	S265<br>S266<br>Z591<br>Z274<br>S294<br>S295	-	-

**HIV**	**DAD**<br>**SDS**<br>**NACRS**	042<br>043<br>044	B20<br>B21<br>B22<br>B23<br>B24	-	-	-	-	-
**Substance use disorder (related to drugs and alcohol use)**	**DAD**<br>**SDS**<br>**NACRS**	V113<br>V6542<br>291<br>292<br>303<br>304<br>3050<br>3052<br>3053<br>3054<br>3055<br>3056<br>357<br>3057<br>3058<br>3059<br>4255<br>5353<br>5710<br>5711<br>5712<br>5713<br>5771<br>6483<br>7903<br>980<br>965<br>967<br>968<br>969<br>E8500<br>E8501<br>E8502<br>E860<br>E9351	1426<br>F10<br>F11<br>F12<br>F13<br>F14<br>F16<br>F18<br>F19<br>G621<br>R780<br>R781<br>R782<br>R783<br>R784<br>R785<br>K70<br>K292<br>K860<br>T40<br>X65<br>Y91<br>Y573<br>Y919<br>Z502<br>Z503<br>Z714<br>Z715<br>Z721<br>Z864	-	-	303<br>304	-	-
	**OMHRS**	**DSM-4/5:** 291, 2920 2929, 303, 3040, 3041, 3042, 30423, 3044, 3045, 3046, 3048, 3050, 3052, 3053, 3054, 3055, 3056, 3057, 3059, 29211, 29212, 29281, 29283, 29284, 29289						

Cirrhosis was defined as a single inpatient cirrhosis code listed above. *Decompensated cirrhosis is defined as having cirrhosis using outpatient cirrhosis code (OHIP:571) and at least one inpatient diagnostic code, procedure code or death code associated with decompensated cirrhosis listed above. HCC was defined as either a diagnosis in the Ontario Cancer Registry or inpatient diagnostic code or death code associated hepatocellular carcinoma as listed above. DAD dataset holds diagnostic and procedural data from inpatient hospital admissions. NACRS dataset holds data on diagnostic and procedural information from ambulatory care and emergency department visits. OHIP dataset holds data on claims made by physicians for universally insured services. OCR is the Ontario Cancer Registry and holds information on all Ontario residents with a cancer diagnosis including diagnosis date and details on the type of cancer. The ORGD dataset holds data on the date and cause of death of Ontario residents. The OMHR dataset holds information on individuals receiving adult mental health services in Ontario, and admissions to mental health–designated hospital beds. *Abbreviations: ICD-9: International Classification of Diseases, 9th revision, ICD-10: International Classification of Diseases, 10th revision; NACRS: National Ambulatory Care Reporting System, OHIP: Ontario Health Insurance Program, DAD: Discharge Abstract Database; OCR: Ontario Cancer Registry; ORGD: Office of the Registrar General- Deaths; OMHRS: Ontario Mental Health Reporting System.*
