# Supplementary material for: Effect of sustained virologic response on liver-related mortality among individuals living with hepatitis C by treatment era: A population-based retrospective cohort study
Source: PLoS One. 2025 Oct 6;20(10):e0333584. doi: 10.1371/journal.pone.0333584 (PMC12500089; doi:10.1371/journal.pone.0333584)
Supplement: S3 Table — (PDF) [file pone.0333584.s003.pdf]

Table S3. Detailed description of diagnostic, death and procedure-related codes

**CIRRHOSIS**		
	**ICD-9 Code**	**ICD-10 Code**
Toxic liver disease with fibrosis and cirrhosis of liver		K.71.7
Alcoholic cirrhosis	571.2	K.70.3
Esophageal varices without bleeding	456.1	I.85.9 , I.98.2
Cirrhosis of the liver w/o alcohol	571.5	K.74.6
**DECOMPENSATED CIRRHOSIS**		
	**ICD-9 Code**	**ICD-10 Code**
Portal hypertension	572.3	K.76.6
Hepatorenal syndrome	572.4	K.76.7
Jaundice	782.4	R.17
Hepatic coma	572.2	-
Hepatic failure	-	K.72.1, K.72.9
Esophageal varices with bleeding	456.0, 456.2	I.85.0, I.98.20, I.98.3
Gastric varices	-	I.86.4
Ascites	789.5	R.18
	**OHIP Code**	
Cirrhosis	571*	
Transjugular intrahepatic portosystemic shunt	J057	
Paracentesis	Z591	
	**Intervention Code (CCI)**	**Procedure Code (CCP)**
Endoscopy for upper GI bleed	1NA1BA-FA, 1NA13BA-X7, 1NA13BA-BD	-
Insertion of Sengstaken tube	-	1006
Transjugular intrahepatic portosystemic shunt	1KQ76.GP-NR	-
Paracentesis	1OT52.HA	6691
Decompensated cirrhosis	**Main cause of death**	**Other Cause of Death**
	5715 (Cirrhosis NOS)<br>5712 (Alcohol-related cirrhosis)<br>5722 (Hepatic coma)<br>5723 (Portal hypertension)<br>5724 (Hepatorenal sx)<br>5728 (Other sequelae chronic liver disease)<br>4560 (Esophageal varices with bleed)	K.72.1 (Chronic hepatic failure)<br>K.72.9 (Hepatic failure, unspecified)<br>K.70.3 (Alcoholic cirrhosis of liver)<br>K.70.4 (Alcoholic hepatic failure)<br>K.71.7 (Toxic liver disease with fibrosis and cirrhosis of liver)<br>K.74 (Fibrosis and cirrhosis of liver)<br>K.74.6 (Other and unspecified cirrhosis of liver)<br>K.76.6 (Portal hypertension)<br>K.76.7 (Hepatorenal syndrome)<br>I.85.X, I982X, I983 (Oesophageal varices) I864 (Gastric varices)
**HEPATOCELLULAR CARCINOMA**		
	**ICD-9 Code**	**ICD-10 Code**
Malignant neoplasm of liver	155.0	C.22.9
Hepatocellular carcinoma	-	C.22.0, 81703
Combined hepatocellular and cholangiocarcinoma	-	81803
	**OCR Code (Morphology)**	**OCR Code (Topography)**
NOS	81703	C220
Scirrhous	81723	
Spindle	81733	
Clear cell	81743	
Pleomorphic	81753	
Combined hepatocellular carcinoma (HCC) and cholangiocarcinoma	81803	
Hepatocellular carcinoma	**Main cause of death**	**Other Cause of Death**
	1550 (Malignant Neoplam of the Liver, Primary)	81703 (HCC NOS)<br>81803 (Combined HCC and cholangiocarcinoma)
**HIV**		
	**ICD-9 Code**	**ICD-10 Code**
Human immunodeficiency virus [HIV] disease	042, 0.43, 0.44	B.20, B.21, B.22, B.23, B.24

**LIVER TRANSPLANT**		
	**ICD-9 Code**	**ICD-10 Code**
Liver replaced by transplant	V.42.7	Z.94.4
Complications of transplanted liver	996.82	T.86.40, T.86.41, T.86.42, T.86.43, T.86.49, T.86.9
	**OHIP Code**	
Living donor, hepatectomy	S265	
Living donor orthotopic liver transplantation recipient	S266	
Donor, liver removal	S274	
Liver excision, liver transplant recipient	S294	
Digestive system-liver, repeat liver transplant	S295	
	**Intervention Code (CCI)**	
Transplant, liver of a deceased donor full size liver	1OA85LAXXK	
Transplant, liver of a deceased donor, multiorgan liver with intestine, pancreas, spleen, or stomach, or any combination of	1OA85VCXXK	
Transplant, liver of a living donor, split liver	1OA85WLXXJ	
Transplant, liver of a deceased donor split liver, or reduced paediatric-size liver	1OA85WLXXK	
**SUBSTANCE USE DISORDER**		
	**ICD-9 Code**	**ICD-10 Code**
Personal history of alcoholism	V.11.3	Z.72.1
Counseling on substance use and abuse	V.65.42	Z.50.2, Z.50.3, Z.71.4, Z.71.5, Y.57.3
Alcohol-induced mental disorders	291	F10
Drug-induced mental disorders	292	F11, F12, F13, F14, F16, F18, F19
Alcohol-dependence syndrome	303	
Drug-dependence	304	Z.86.4
Abuse of alcohol cannabis, hallucinogen, sedative, opioid, cocaine, amphetamine or related acting sympathomimetic, antidepressant type, Other, mixed, or unspecified drug abuse	305.0, 305.2, 305.3, 305.4, 305.5, 305.6, 305.7, 305.8, 305.9	
Alcoholic polyneuropathy	357.5	G.62.1
Alcoholic cardiomyopathy	425.5	I.42.6
Alcoholic gastritis	535.3	K.29.2
Alcohol-induced chronic pancreatitis	577.1	K.86.0
Alcoholic fatty liver, hepatitis, cirrhosis liver damage	571.0, 571.1, 571.2, 571.3	K.70
Drug dependence in the mother, but complicating pregnancy, childbirth, or the puerperium	648.3	
Excess blood-alcohol level and finding of opiate drug, cocaine, hallucinogen, psychotropic other drugs of addictive potential.	790.3	R.78.0, R781, R782 R783, R784, R785
Toxic effect of alcohol	980	
Poisoning by analgesics, antipyretics, and antirheumatics, poisoning by sedatives and hypnotics, poisoning by other central nervous system depressants and anesthetics, poisoning by psychotropic agents	965, 967, 968, 969	T.40, X.65, Y.91
Accidental poisoning - heroin, methadone, opiates	E8500, E8501, E8502	
Accidental poisoning - alcohol	E860	
Adverse effects of methadone	E9351	
	**DSM4/ DSM5**	
Alcohol intoxication or withdrawal, delirium	291	
Substance, sedative, hypnotic or anxiolytic withdrawal	2920	
Substance-related disorder NOS	2929	
Alcohol intoxication/ dependence	303	
Dependence – opioid, sedative, cocaine, cannabis, amphetamine, hallucinogen, and others	3040, 3041, 3042, 3043, 3044, 3045, 3046, 3048	
Substance abuse – opioid, sedative, cocaine, cannabis, amphetamine, hallucinogen, and others	3050, 3052, 3053, 3054, 3055, 3056, 3057, 3059	
Substance-induced psychotic disorder	29211, 29212	
Substance intoxication delirium	29281	
Substance-induced persisting amnestic disorder	29283	

Cirrhosis was defined as a single inpatient cirrhosis code listed above. *Decompensated cirrhosis is defined as having cirrhosis using outpatient cirrhosis code (OHIP:571) and at least one inpatient diagnostic code, procedure code or death code associated with decompensated cirrhosis listed above. *Abbreviations: ICD-9: International Classification of Diseases, 9th revision, ICD-10: International Classification of Diseases, 10th revision; OHIP: Ontario Health Insurance Program, OCR: Ontario Cancer Registry.*
