# Supplementary material for: Effect of sustained virologic response on liver-related mortality among individuals living with hepatitis C by treatment era: A population-based retrospective cohort study
Source: PLoS One. 2025 Oct 6;20(10):e0333584. doi: 10.1371/journal.pone.0333584 (PMC12500089; doi:10.1371/journal.pone.0333584)
Supplement: S4 Table — (PDF) [file pone.0333584.s004.pdf]

Table S4. Death code used to identify liver-related death

LIVER DEATHS	Main cause of death (liver-related)	Other cause of death (liver-related)
	5715 (Cirrhosis NOS)<br>5712 (Alcohol-related cirrhosis)<br>5722 (Hepatic coma)<br>5723 (Portal hypertension)<br>5724 (Hepatorenal sx)<br>5728 (Other sequelae chronic liver disease)<br>4560 (Esophageal varices with bleed)<br>1550 (Malignant Neoplasm of the Liver, Primary)	K.72.1 (Chronic hepatic failure)<br>K.72.9 (Hepatic failure, unspecified)<br>K.70.3 (Alcoholic cirrhosis of liver)<br>K.70.4 (Alcoholic hepatic failure)<br>K.71.7 (Toxic liver disease with fibrosis and cirrhosis of liver)<br>K.74 (Fibrosis and cirrhosis of liver)<br>K.74.6 (Other and unspecified cirrhosis of liver)<br>K.76.6 (Portal hypertension)<br>K.76.7 (Hepatorenal syndrome)<br>I.85.X, I982X, I983 (Oesophageal varices)<br>I864 (Gastric varices)
	Deaths associated with Ontario Cancer Registry Codes 81703 (HCC NOS) and 81803 (Combined HCC and cholangiocarcinoma)	

Abbreviations: HCC: Hepatocellular carcinoma.
