# Supplementary material for: Effect of sustained virologic response on liver-related mortality among individuals living with hepatitis C by treatment era: A population-based retrospective cohort study
Source: PLoS One. 2025 Oct 6;20(10):e0333584. doi: 10.1371/journal.pone.0333584 (PMC12500089; doi:10.1371/journal.pone.0333584)
Supplement: S5 Table — (PDF) [file pone.0333584.s005.pdf]

Table S5. Characteristics of study cohort stratified by liver disease severity and treatment era

		TREATMENT ERA			TREATMENT ERA			TREATMENT ERA	
	No Cirrhosis	Pre-DAA [1999-2013]	DAA [2014-2018]	Compensated Cirrhosis	Pre-DAA [1999-2013]	DAA [2014-2018]	Advanced Liver Disease	Pre-DAA [1999-2013]	DAA [2014-2018]
	N=57,568	N=41,590	N=15,978	N=5,058	N=4,051	N=1,007	N=10,785	N=9,213	N=1,572
**Age in years, mean (SD)**									
Age at diagnosis	43.0 (12.9)	43.3 (12.2)	42.1 (14.4)	48.9 (11.6)	47.9 (10.9)	53.0 (13.3)	51.9 (10.7)	51.3 (10.3)	55.3 (12.1)
**Birth cohort, N (%)**									
<1945	3,247 (5.6)	2,801 (6.7)	446 (2.8)	522 (10.3)	447 (11.0)	75 (7.4)	1,744 (16.2)	1,593 (17.3)	151 (9.6)
1945-1965	30,231 (52.5)	24,330 (58.5)	5,901 (36.9)	3,564 (70.5)	2,942 (72.6)	622 (61.8)	8,065 (74.8)	6,932 (75.2)	1,133 (72.1)
>1965	24,090 (41.9)	14,459 (34.8)	9,631 (60.3)	972 (19.2)	662 (16.4)	310 (30.8)	976 (9.0)	688 (7.5)	288 (18.3)
**Male sex, N (%)**	36,664 (63.8)	26,538 (63.9)	10,126 (63.5)	3,381 (66.8)	2,682 (66.2)	699 (69.4)	7,711 (71.5)	6,561 (71.2)	1,150 (73.2)
**Rural, N (%)**	5,939 (10.5)	3,778 (9.2)	2,161 (13.7)	471 (9.4)	359 (8.9)	112 (11.2)	1,058 (9.9)	873 (9.5)	185 (11.9)
**Neighborhood income quintile, N (%)**									
Low (quintiles 1-2)	32,988 (58.6)	23,273 (57.3)	9,715 (62.0)	2,690 (53.6)	2,124 (52.8)	566 (56.8)	5,845 (54.7)	4,965 (54.4)	880 (56.5)
Medium (quintile 3)	9,678 (17.2)	7,126 (17.5)	2,552 (16.3)	927 (18.5)	742 (18.5)	185 (18.6)	1,905 (17.8)	1,629 (17.8)	276 (17.7)
High (quintiles 4-5)	13,632 (24.2)	10,240 (25.2)	3,392 (21.7)	1,401 (27.9)	1,155 (28.7)	246 (24.6)	2,936 (27.5)	2,535 (27.8)	401 (25.8)
**Residential instability quintile, N (%)**									
Low (quintiles 1-2)	12,741 (23.2)	9,610 (24.2)	3,131 (20.8)	1,322 (26.7)	1,081 (27.3)	241 (24.4)	2,841 (27.1)	2,478 (27.6)	363 (23.8)
Medium (quintiles 3)	8,583 (15.6)	6,258 (15.7)	2,325 (15.4)	849 (17.2)	673 (17.0)	176 (17.8)	1,808 (17.2)	1,556 (17.3)	252 (16.5)
High (quintiles 4-5)	33,525 (61.2)	23,913 (60.1)	9,612 (63.8)	2,779 (56.1)	2,208 (55.7)	571 (57.8)	5,846 (55.7)	4,938 (55.1)	908 (59.7)
**Material deprivation quintile, N (%)**									
Low (quintiles 1-2)	13,439 (24.5)	9,960 (25.0)	3,479 (23.1)	1,394 (28.2)	1,144 (28.9)	250 (25.3)	2,847 (27.1)	2,444 (27.2)	403 (26.5)
Medium (quintile 3)	9,312 (17.0)	6,873 (17.3)	2,439 (16.2)	875 (17.7)	682 (17.2)	193 (19.5)	1,877 (17.9)	1,606 (17.9)	271 (17.8)
High (quintiles 4-5)	32,098 (58.5)	22,948 (57.7)	9,150 (60.7)	2,681 (54.1)	2,136 (53.9)	545 (55.2)	5,771 (55.0)	4,922 (54.9)	849 (55.7)
**Ethnic concentration quintile, N (%)**									
Low (quintiles 1-2)	19,921 (36.3)	13,576 (34.1)	6,345 (42.1)	1,658 (33.5)	1,268 (32.0)	390 (39.5)	3,626 (34.5)	3,020 (33.7)	606 (39.8)
Medium (quintile 3)	10,099 (18.4)	7,102 (17.9)	2,997 (19.9)	945 (19.1)	746 (18.8)	199 (20.1)	1,898 (18.1)	1,613 (18)	285 (18.7)
High (quintiles 4-5)	24,829 (45.3)	19,103 (48.0)	5,726 (38.0)	2,347 (47.4)	1,948 (49.2)	399 (40.4)	4,971 (47.4)	4,339 (48.3)	632 (41.5)
**Immigrant N (%)**	7,148 (12.4)	5,744 (13.8)	1,404 (8.8)	498 (9.8)	399 (9.8)	99 (9.8)	1,197 (11.1)	1,067 (11.6)	130 (8.3)
**Substance use disorder (ever), N (%)**	28,277 (49.1)	18,581 (44.7)	9,696 (60.7)	2,123 (42.0)	1,644 (40.6)	479 (47.6)	5,978 (44.6)	4,996 (54.2)	982 (62.5)
**HIV positivity, N (%)**	705 (1.2)	582 (1.4)	123 (0.8)	55 (1.1)	49 (1.2)	6 (0.6)	140 (1.3)	129 (1.4)	11 (0.7)
**HBV antigen positivity*, N (%)**	345 (0.6)	266 (0.6)	79 (0.5)	36 (0.7)	28 (0.7)	8 (0.8)	82 (0.8)	64 (0.7)	18 (1.1)
**Aggregated diagnosis group categories, N (%)**									
0-3 ADGs	21,799 (37.9)	15,639 (37.7)	6,160 (38.6)	1,400 (27.7)	1,082 (26.7)	318 (31.6)	2,907 (27.0)	2,436 (26.4)	471 (30)
4-7 ADGs	22,736 (39.6)	16,741 (40.3)	5,995 (37.6)	2,114 (41.8)	1,711 (42.2)	403 (40.0)	4,495 (41.7)	3,899 (42.3)	596 (37.9)
8-10 ADGs	8,457 (14.7)	6,093 (14.7)	2,364 (14.8)	956 (18.9)	787 (19.4)	169 (16.8)	2,049 (19.0)	1,763 (19.1)	286 (18.2)
>11 ADGs	4,488 (7.8)	3,062 (7.3)	1,426 (9.0)	588 (11.6)	471 (11.7)	117 (11.6)	1,334 (12.3)	1,115 (12.2)	219 (13.9)
**Liver disease severity at diagnosis, N (%)**									
Non-cirrhotic (NC)	57,568 (100)	41,590 (100)	15,978 (100)	35,058 (100)	-	-	-	-	-
Compensated cirrhosis (CC)	-	-	-	-	4,051 (100)	1,007 (100)	-	-	-
Decompensated cirrhosis (DC)	-	-	-	-	-	-	7,533 (69.8)	6,477 (70.3)	1,056 (67.2)
Hepatocellular carcinoma (HCC)	-	-	-	-	-	-	3,252 (30.2)	2,736 (29.7)	516 (32.8)
**Liver transplant, N (%)**	0 (.)	0 (.)	0 (.)	0 (.)	0 (.)	0 (.)	994 (9.2)	782 (8.5)	212 (13.5)
**HCV genotype, N (%)**									
Genotype 1	33,324 (63.2)	24,355 (64.0)	8,969 (61.2)	3,123 (61.7)	2,520 (67.7)	603 (63.1)	6,821 (68.0)	5,905 (69)	916 (61.9)
Genotype 2	5,867 (11.1)	4,676 (12.3)	1,191 (8.1)	595 (11.8)	499 (13.4)	96 (10.1)	929 (9.3)	783 (9.2)	146 (9.9)
Genotype 3	11,289 (21.4)	7,526 (19.8)	3,763 (25.7)	781 (15.4)	582 (15.6)	199 (20.8)	1,780 (17.7)	1,468 (17.2)	312 (21.1)
Genotype 4	1,008 (1.9)	799 (2.1)	209 (1.4)	91 (1.8)	67 (1.8)	24 (2.5)	285 (2.8)	266 (3.1)	19 (1.3)
**Treated, N (%)**	27,129 (47.1)	14,205 (34.2)	6,376 (39.9)	2,707 (53.5)	1,662 (41.0)	508 (50.4)	5,096 (47.3)	3,180 (34.5)	687 (43.7)
**SVR, N (% of treated)**	22,056 (81.3)	11,551 (81.3)	5,170 (81.1)	2,216 (83.4)	1,281 (77.1)	487 (95.9)	3,793 (74.7)	1,900 (59.7)	612 (89.1)

Baseline characteristics of the study cohort stratified by liver disease severity at the time of HCV RNA diagnosis for all individuals and for those diagnosed during the pre-DAA era (Jan 1999- Dec 2013) and DAA era (Jan 2014- Dec 2018). Frequencies are calculated after exclusion of missing values. *HBV diagnosis is based on hepatitis b surface antigen (HBsAg) reactivity. *Abbreviations: ADG: aggregated diagnostic groups; CC: compensated cirrhosis, DAA: direct-acting antiviral; DC: decompensated cirrhosis; HBV: hepatitis B virus; HCC: hepatocellular carcinoma, HCV: hepatitis C virus; HIV: human immunodeficiency virus; NC: no cirrhosis; N: number of observations; q: quintile; SD: standard deviation; SVR: sustained viral response*
