# Supplementary material for: Effect of sustained virologic response on liver-related mortality among individuals living with hepatitis C by treatment era: A population-based retrospective cohort study
Source: PLoS One. 2025 Oct 6;20(10):e0333584. doi: 10.1371/journal.pone.0333584 (PMC12500089; doi:10.1371/journal.pone.0333584)
Supplement: S6 Table — (PDF) [file pone.0333584.s006.pdf]

Table S6. Characteristics of study cohort stratified by substance use and treatment era

	No Substance Use Disorder	TREATMENT ERA Pre-DAA [1999-2013]	TREATMENT ERA DAA [2014-2018]	Substance Use Disorder	TREATMENT ERA Pre-DAA [1999-2013]	TREATMENT ERA DAA [2014-2018]
	N=37,033	N=29,633	N=7,400	N=36,378	N=25,221	N=11,157
**Age in years, mean (SD)**						
Age at diagnosis	48.3 (13.4)	47.6 (12.9)	50.9 (15.0)	41.0 (11.3)	41.9 (10.6)	39.1 (12.7)
**Birth cohort, N (%)**						
<1945	4,702 (12.7)	4,115 (13.9)	587 (7.9)	811 (2.2)	726 (2.9)	85 (0.8)
1945-1965	22,982 (62.1)	18,796 (63.4)	4,186 (56.6)	18,878 (51.9)	15,408 (61.1)	3,470 (31.1)
>1965	9,349 (25.2)	6,722 (22.7)	2,627 (35.5)	16,689 (45.9)	9,087 (36.0)	7,602 (68.1)
**Male sex, N (%)**	22,807 (61.7)	18,111 (61.2)	4,696 (63.7)	24,949 (68.6)	17,670 (70.1)	7,279 (65.2)
**Rural, N (%)**	3,201 (8.8)	2,401 (8.3)	800 (11.0)	31,795 (88.2)	2,609 (10.4)	1,658 (15.0)
**Neighborhood income quintile, N (%)**						
Low (quintiles 1-2)	18,830 (52.0)	14,883 (51.4)	3,947 (54.5)	22,693 (63.3)	15,479 (62.3)	7,214 (65.7)
Medium (quintile 3)	6,851 (18.9)	5,494 (19.0)	1,357 (18.7)	5,659 (15.8)	4,003 (16.1)	1,656 (15.1)
High (quintiles 4-5)	10,499 (29.1)	8,565 (29.6)	1,934 (26.8)	7,470 (20.9)	5,365 (21.6)	2,105 (19.2)
**Residential instability quintile, N (%)**						
Low (quintiles 1-2)	10,982 (30.8)	8,942 (31.3)	2,040 (28.7)	5,922 (17.1)	4,227 (17.5)	1,695 (16.2)
Medium (quintile 3)	6,071 (17.0)	4,851 (17.0)	1,220 (17.1)	5,169 (14.9)	3,636 (15.1)	1,533 (14.7)
High (quintiles 4-5)	18,621 (52.2)	14,763 (51.7)	3,858 (54.2)	23,529 (68.0)	16,296 (67.4)	7,233 (69.1)
**Material deprivation quintile, N (%)**						
Low (quintiles 1-2)	10,545 (29.6)	8,510 (29.8)	2,035 (28.6)	7,135 (20.6)	5,038 (20.9)	2,097 (20.0)
Medium (quintile 3)	6,528 (18.3)	5,243 (18.4)	1,285 (18.1)	5,536 (16.0)	3,918 (16.2)	1,618 (15.5)
High (quintiles 4-5)	18,601 (52.1)	14,803 (51.8)	3,798 (53.3)	21,949 (63.4)	15,203 (62.9)	6,746 (64.5)
**Ethnic concentration quintile, N (%)**						
Low (quintiles 1-2)	10,854 (30.4)	8,359 (29.3)	2,495 (35.1)	14,351 (41.5)	9,505 (39.3)	4,846 (46.3)
Medium (quintile 3)	5,979 (16.8)	4,703 (16.5)	1,276 (17.9)	6,963 (20.1)	4,758 (19.7)	2,205 (21.1)
High (quintiles 4-5)	18,841 (52.8)	15,494 (54.2)	3,347 (47.0)	13,306 (38.4)	9,896 (41.0)	3,410 (32.6)
**Immigrant N (%)**	7,858 (21.2)	6,476 (21.9)	1,382 (18.7)	985 (2.7)	734 (2.9)	251 (2.2)
**HIV positivity, N (%)**	331 (0.9)	285 (1.0)	46 (0.6)	569 (1.6)	475 (1.9)	94 (0.8)
**HBV antigen positivity*, N (%)**	286 (0.8)	229 (0.8)	57 (0.8)	177 (0.5)	129 (0.5)	48 (0.4)
**Aggregated diagnosis group categories, N (%)**						
0-3 ADGs	15,879 (43.0)	12,225 (41.3)	3,654 (49.6)	10,227 (28.1)	6,932 (27.5)	2,679 (28.9)
4-7 ADGs	14,922 (40.4)	12,272 (41.5)	2,650 (36.0)	14,423 (39.6)	10,079 (40)	3,665 (39.6)
8-10 ADGs	4,435 (12.0)	3,690 (12.5)	745 (10.1)	7,027 (19.3)	4,953 (19.6)	1,712 (18.5)
>11 ADGs	1,709 (4.6)	1,391 (4.7)	318 (4.3)	4,701 (13.0)	3,257 (12.9)	1,206 (13.0)
**Liver disease severity at diagnosis, N (%)**						
Non-cirrhotic (NC)	29,291 (79.1)	23,009 (77.6)	6,282 (84.9)	28,277 (77.7)	20,198 (74.5)	9,696 (86.9)
Compensated cirrhosis (CC)	2,935 (7.9)	2,407 (8.1)	528 (7.1)	2,123 (5.8)	1,715 (6.3)	479 (4.3)
Decompensated cirrhosis (DC)	3,076 (8.3)	2,745 (9.3)	331 (4.5)	4,457 (12.3)	3,881 (14.3)	725 (6.5)
Hepatocellular carcinoma (HCC)	1,731 (4.7)	1,472 (5.0)	259 (3.5)	1,521 (4.2)	1,322 (4.9)	257 (2.3)
**Liver transplant, N (%)**	438 (1.2)	361 (1.2)	77 (1.0)	556 (1.5)	447 (1.6)	135 (1.2)
**HCV genotype, N (%)**						
Genotype 1	21,362 (63.0)	17,176 (63.5)	4,186 (61.3)	21,906 (65.3)	15,604 (67.0)	6,302 (61.5)
Genotype 2	4,386 (12.9)	3,596 (13.3)	790 (11.6)	3,005 (9.0)	2,362 (10.1)	643 (6.3)
Genotype 3	5,937 (17.5)	4,662 (17.2)	1,275 (18.7)	7,913 (23.6)	4,914 (21.1)	2,999 (29.3)
Genotype 4	1,221 (3.6)	1,003 (3.7)	218 (3.2)	163 (0.5)	129 (0.6)	34 (0.3)
Other/mixed	988 (2.9)	625 (2.3)	363 (5.3)	546 (1.6)	275 (1.2)	271 (2.6)
**Treated, N (%)**	19,229 (51.9)	11,829 (39.9)	3,478 (47.0)	15,703 (43.2)	7,218 (28.6)	4,093 (35.5)
**SVR, N (% of treated)**	16,629 (86.5)	9,622 (81.3)	3,342 (96.1)	11,481 (73.1)	5,190 (71.9)	2,927 (71.5)

Baseline characteristics of the study cohort at the time of HCV RNA diagnosis stratified by substance use for all individuals and for those diagnosed during the pre-DAA era (Jan 1999- Dec 2013) and DAA era (Jan 2014- Dec 2018). Frequencies are calculated after exclusion of missing values. *HBV diagnosis is based on hepatitis b surface antigen (HBsAg) reactivity. *Abbreviations: ADG: aggregated diagnostic groups; CC: compensated cirrhosis, DAA: direct-acting antiviral; DC: decompensated cirrhosis; HBV: hepatitis B virus; HCC: hepatocellular carcinoma, HCV: hepatitis C virus; HIV: human immunodeficiency virus; NC: no cirrhosis; N: number of observations; q: quintile; SD: standard deviation; SVR: sustained viral response*
