# Supplementary material for: Effect of sustained virologic response on liver-related mortality among individuals living with hepatitis C by treatment era: A population-based retrospective cohort study
Source: PLoS One. 2025 Oct 6;20(10):e0333584. doi: 10.1371/journal.pone.0333584 (PMC12500089; doi:10.1371/journal.pone.0333584)
Supplement: S7 Table — (PDF) [file pone.0333584.s007.pdf]

Table S7. Incidence of clinical events in the study cohort by SVR achievement

		Liver Disease Severity at Diagnosis				
	**Study Cohort**	**No Cirrhosis**	**Compensated cirrhosis**	**Advance Liver Disease**	**No Substance Use Disorder**	**Substance Use Disorder**
	IR per 1,000 PY (95%CI)	IR per 1,000 PY (95%CI)	IR per 1,000 PY (95%CI)	IR per 1,000 PY (95%CI)	IR per 1,000 PY (95%CI)	IR per 1,000 PY (95%CI)
**Non-liver-related death**	4.0 (3.8-4.1)	1.1 (1.0-1.1)	2.9 (2.5-3.4)	22.4 (21.5-23.4)	3.3 (3.1-3.5)	4.8 (4.5-5.0)
No SVR	5.9 (5.7-6.2)	1.7 (1.6-1.8)	5.3 (4.6-6.2)	34.7 (33.1-36.3)	5.3 (5.0-5.6)	6.6 (6.3-7.0)
SVR	1.5 (1.3-1.6)	0.2 (0.2-0.3)	0.5 (0.3-0.8)	9.0 (8.2-9.9)	1.2 (1.1-1.4)	1.8 (1.6-2.1)
**Non-liver-related death**	17.4 (17.2-17.7)	13.9 (13.6-14.2)	15.5 (14.5-16.5)	40.3 (39.0-41.6)	11.6 (11.2-11.9)	24.8 (24.3-25.3)
No SVR	25.6 (25.1-26.1)	20.1 (19.7-20.6)	25.3 (23.6-27.2)	62.2 (60.1-64.4)	18.6 (18.0-19.1)	32.8 (32.1-33.6)
SVR	7.2 (6.9-7.5)	5.8 (5.5-6.1)	5.6 (4.8-6.5)	16.3 (15.2-17.5)	4.3 (4.1-4.6)	11.8 (11.3-12.5)
**All-cause death**	21.4 (21.1-21.7)	15.0 (14.7-15.3)	18.4 (17.4-19.6)	62.7 (61.2-64.3)	14.9 (14.5-15.2)	29.6 (29.0-30.2)
No SVR	31.5 (31.0-32.4)	21.8 (21.3-22.3)	30.6 (28.7-32.7)	96.9 (94.2-99.6)	23.9 (23.2-24.5)	39.4 (35.8-40.2)
SVR	8.7 (8.4-8.9)	6.0 (5.7-6.3)	6.0 (5.2-7.0)	25.4 (24.0-26.8)	5.6 (5.3-5.9)	13.7 (13.0-14.3)

Incidence of clinical events per 1,000 person-years in the study cohort for those with and without SVR stratified by liver disease severity at the time of HCV RNA diagnosis and by the presence of absence of substance use disorder. Predicted incidence rates were estimated using Poisson regression. *Abbreviations: CI: confidence interval; IR: incidence rate; PY: person-years; SVR: sustained viral response.*
