# Supplementary material for: Effect of sustained virologic response on liver-related mortality among individuals living with hepatitis C by treatment era: A population-based retrospective cohort study
Source: PLoS One. 2025 Oct 6;20(10):e0333584. doi: 10.1371/journal.pone.0333584 (PMC12500089; doi:10.1371/journal.pone.0333584)
Supplement: Attachment — Submitted filename: Response to Reviewers.docx [file pone.0333584.s009.docx]

Dear Editor

Thank you for the opportunity to re-submit our revised manuscript, “Effect of sustained viral clearance on liver-related mortality among individuals living with hepatitis C by treatment era: a population-based retrospective cohort study” (**PONE-D-25-05248**) for publication in the *PLOS One.*

We thank the Reviewers for their constructive feedback and insightful suggestions that helped us to improve the quality and clarity of our work. Please see below our detailed response to each of the comments. The changes are tracked in the revised manuscript. We are also submitting the clean copy, as requested.

We also noticed that Tables 1 and 2 were mistakenly duplicated in the original submission. This has been corrected, and the proper Table 1 has now been included.

Once again, we express our appreciation for your detailed review and consideration. Please kindly let us know if there are any further suggestions or comments.

Sincerely,

Beate Sander

Senior Scientist

University Health Network

200 Elizabeth Street, Toronto, ON M5G 2C4

E-mail: [beate.sander@uhn.ca](mailto:beate.sander@uhn.ca)

**EDITOR COMMENTS**

1. **Please ensure that your manuscript meets PLOS ONE's style requirements, including those for file naming.**

*Response:* Thank you. The manuscript has been formatted per the PLOS ONE style templates.

2. **We note that the grant information you provided in the ‘Funding Information’ and ‘Financial Disclosure’ sections do not match.**

*Response:* Thank you for your comment. We have revised the Funding Information and now both sections match.

**When you resubmit, please ensure that you provide the correct grant numbers for the awards you received for your study in the ‘Funding Information’ section.**

*Response:* We have reviewed the ‘Funding Information’ section and the grant numbers appear to be correct. Please let us know if any additional clarification is needed.

**3. Thank you for stating the following in the Competing Interests section:**

**“Dr. W. Wong reports a grant from Canadian Liver Foundation, outside the submitted work.”**

**Please confirm that this does not alter your adherence to all PLOS ONE policies on sharing data and materials, by including the following statement: "This does not alter our adherence to  PLOS ONE policies on sharing data and materials.” (as detailed online in our guide for authors** [**http://journals.plos.org/plosone/s/competing-interests**](https://urldefense.com/v3/__http:/journals.plos.org/plosone/s/competing-interests__;!!CjcC7IQ!NmPW3YZqQ-jHRbnnGF0-6y6fOT6eVCPIoP-8b1GuFMPfbE8Y2tzxckiWeyuNAeN3FrLmseFHYwyOGVyl0sQ7WKs$)**).  If there are restrictions on sharing of data and/or materials, please state these. Please note that we cannot proceed with consideration of your article until this information has been declared.**

**Please include your updated Competing Interests statement in your cover letter; we will change the online submission form on your behalf.**

*Response:* Thank you. We have added a revised Competing Interest Statement in the cover letter.

**4. We note that you have indicated that there are restrictions to data sharing for this study. For studies involving human research participant data or other sensitive data, we encourage authors to share de-identified or anonymized data. However, when data cannot be publicly shared for ethical reasons, we allow authors to make their data sets available upon request. For information on unacceptable data access restrictions, please see** [**http://journals.plos.org/plosone/s/data-availability#loc-unacceptable-data-access-restrictions**](https://urldefense.com/v3/__http:/journals.plos.org/plosone/s/data-availability*loc-unacceptable-data-access-restrictions__;Iw!!CjcC7IQ!NmPW3YZqQ-jHRbnnGF0-6y6fOT6eVCPIoP-8b1GuFMPfbE8Y2tzxckiWeyuNAeN3FrLmseFHYwyOGVyllfzTKaQ$)**.**

**Before we proceed with your manuscript, please address the following prompts:**

**If there are ethical or legal restrictions on sharing a de-identified data set, please explain them in detail (e.g., data contain potentially identifying or sensitive patient information, data are owned by a third-party organization, etc.) and who has imposed them (e.g., a Research Ethics Committee or Institutional Review Board, etc.). Please also provide contact information for a data access committee, ethics committee, or other institutional body to which data requests may be sent.**

**Please update your Data Availability statement in the submission form accordingly.**

*Response:* We have updated the Data Availability statement and added that data are owned by a third-party organization. The full statement is below.

***Data Availability:*** *Data for this study are owned by a third-party organization and is held securely in coded form at ICES. While legal data sharing agreements between ICES and data providers (e.g., healthcare organizations and government) prohibit ICES from making the dataset publicly available, access may be granted to those who meet pre-specified criteria for confidential access, available at www.ices.on.ca/DAS (email: das@ices.on.ca). The full dataset creation plan and underlying analytic code are available from the authors upon request, understanding that the computer programs may rely upon coding templates or macros that are unique to ICES and are therefore either inaccessible or may require modification.*

**REVIEWERS' COMMENTS:**

**REVIEWER #1:**

**This article is covering an important topic on HCV. Unfortunately it is not easily readable, especially the abstract and introduction is not very attractive for the reader. I do not see much impact of this article to the future. On the other hand many known or expected facts have been confirmed.**

*Response:* Thank you for your feedback. We appreciate your comments regarding the importance of the topic. This study uses a real-world, population-level data from Ontario, encompassing a very large sample size, which enhances the generalizability and validity of the findings. We acknowledge that the abstract and introduction could be clearer and more engaging, and have revised both sections (please see below) to improve readability and better highlight the relevance and potential implications of our findings.

**Abstract**

**Introduction and Objectives:** Sustained virologic response (SVR) is a validated surrogate marker for successful hepatitis C virus (HCV) treatment. Historically, interferon-based therapies, the standard of care for decades, offered only limited efficacy with respect to SVR. The recent introduction of highly effective direct-acting antivirals (DAAs) revolutionised treatment, expanding treatment eligibility among individuals with advanced liver disease (ALD) and drug/alcohol-related substance use disorder. Given these clinical policy shifts, we assessed the real-world impact of SVR on liver-related death for these key clinical groups for whom treatment had previously been less feasible.

**Materials and Methods:** We conducted a population-based, cohort study of Ontario residents with HCV viremia between January 1st, 1999, and December 31st, 2018, with follow-up to May 31st, 2021 (N=73,411) and used cause-specific hazard models to explore the association between SVR and liver-related death.

**Results**: SVR was associated with a significant reduction in liver-related deaths (adjusted hazard ratio [aHR]: 0.22, 95%CI: 0.20-0.24). This benefit was consistent across all levels of liver disease severity, including individuals with (aHR: 0.11, 95%CI: 0.06-0.17) and without (aHR: 0.13, 95%CI: 0.10-0.17) cirrhosis, individuals with ALD (aHR: 0.24, 95%CI: 0.22-0.27) as well as among individuals with (aHR: 0.24, 95%CI: 0.21-0.27) and without (aHR: 0.21, 95%CI: 0.18-0.24) substance use disorder.

**Conclusions:** This study demonstrates the real-world impact of SVR on liver-related mortality and highlights the value of early treatment and continued support for populations who are marginalised.

**INTRODUCTION:**

Chronic hepatitis C (CHC) is associated with major morbidity and mortality. Over time, the chronic persistence of hepatitis C virus (HCV) can cause progressive liver damage, ranging from hepatic fibrosis to cirrhosis, and end-stage liver disease, often requiring liver transplantation or resulting in liver-related mortality [1-3].

Sustained virologic response (SVR) is a clinically relevant surrogate endpoint in HCV treatment, defined as the absence of detectable HCV RNA in the blood 12 or 24 weeks after completing antiviral therapy. Indeed, the achievement of SVR has been shown to substantially reduce mortality for individuals living with hepatitis C [4, 5]. However, for decades the standard-of-care for HCV consisted of interferon-based therapy, offering only limited efficacy with respect to SVR [5,6]. Moreover, interferon-based therapies were of limited use for individuals with a history of substance use and mental illness due to poor tolerability and neuropsychiatric side effects [6]. Similarly, tolerability was also a concern for those with advanced liver disease (ALD) [7]. In fact, interferon-based regiments were contraindicated for those with decompensated cirrhosis (DC) and demonstrated only limited efficacy in treating recurrent infection among immunosuppressed liver-transplant recipients [7, 8].

Since the introduction of highly effective direct-acting antivirals (DAAs) in 2013, clinical policy guidelines for CHC have been rapidly changing. DAAs are significantly more effective and better tolerated, enabling treatment for individuals who were previously ineligible, including those with ALD and comorbid conditions such as substance use. [9-12]. Moreover, compared to earlier therapies, DAAs demonstrate superior outcomes among post-transplant individuals [8, 13], and eliminate the need for genotype-specific regimens due to their pan-genotypic activity [14]. Finally, the high rates of SVR exhibited by DAAs also offer an opportunity to eliminate HCV as a public health concern and meet the WHO target of a 65% reduction in mortality by 2030 [15]. However, despite rapid shifts in clinical practice and public health policy, real-world impact of SVR on long-term outcome such as liver-related death in the DAA era, specifically among higher-risk populations is lacking.

Our primary objective was to assess the real-world impact of SVR on liver-related death in the DAA and pre-DAA treatment eras among important clinical population such as those with advanced liver disease and substance use and our secondary objective was to explore changes in predictors of liver deaths by treatment era from a population-based cohort of individuals living with hepatitis C in the province of Ontario, Canada’s largest jurisdiction.

**Other comments and questions concerning specific part of the text which should be corrected or explained:
- line 40+397: Do you mean liver-related mortality?**

*Response:* Thank you for the clarification. Yes, we were referring to liver-related mortality, and the wording has been revised accordingly.

**- SVR was mentioned several times before you explained it (line 107) and the abbreviation is translated in two different ways in the article (sustained viral clearance - most cases and sustained virologic response- line 289 and 311). I don't think sustained viral clearance is the correct term because both European and American guidelines use the term sustained virologic response.**

*Response:* Thank you for pointing this out. We have corrected the terminology [SVR: sustained virologic response] throughout the manuscript.

We have also added an explanation of SVR in the *Introduction* section, where we first introduced the term. “*Sustained virologic response (SVR) is a clinically relevant surrogate endpoint in HCV treatment, defined as the absence of detectable HCV RNA in the blood 12 or 24 weeks after completing antiviral therapy*.”

We kept the definition in line 107, where we describe how individuals with SVR were identified based on available data and existing data sources.

**- line 98 and further: ICES - Is there any explanation of this abbreviation?**

*Response:* In 2018, the institute formerly known as the Institute for Clinical Evaluative Sciences formally adopted the initialism ICES as its official name. We added this explanation in the *Study population and setting section.*

*“After the exclusion of individuals (N=6,488) whose laboratory testing records could not be linked to demographic data of all Ontario residents held at ICES (formerly known as the Institute for Clinical Evaluative Sciences), our study cohort included 73,411 unique individuals with confirmed HCV viremia.”*

**- line 156+165: I would suggest HCV RNA positivity instead of RNA diagnosis**

*Response:* Thank you for the suggestion. We agree and have revised the wording throughout the manuscript.

**- line 160-161: Why 5 % of patients had HCC at diagnosis if almost 80 % did not have cirrhosis? How was the cirrhosis diagnosis made?**

*Response:* Thank you for your question. These findings are based on population-level administrative data for the entire Ontario population. We identified both HCC and cirrhosis from datasets held at ICES using validated diagnostic algorithms and diagnostic codes, which are provided in the Supplementary Tables S2 and S3. Compensated cirrhosis, decompensated cirrhosis, and hepatocellular carcinoma (HCC) were treated as mutually exclusive conditions.

We also noticed that Tables 1 and 2 were mistakenly duplicated in the original submission. This has been corrected, and the appropriate Table 1, depicting the baseline characteristics of the cohort at the time of RNA positivity, has now been included (see also below).

**Table 1. Demographic characteristics of study cohort stratified by treatment era**

|  |  | **TREATMENT ERA** | |
| --- | --- | --- | --- |
|  | **Study Cohort N=73,411** | **PRE-DAA N=54,854** | **DAA N=18,557** |
| **Age in years, mean (SD)** |  |  |  |
| At time of diagnosis | 44.7 (12.9) | 45.0 (12.2) | 43.8 (14.8) |
| **Birth year, N (%)** |  |  |  |
| <1945 | 5,513 (7.5) | 4,841 (8.8) | 672 (3.6) |
| 1945-1965 | 41,860 (57.0) | 34,204 (62.4) | 7,656 (41.3) |
| >1965 | 26,038 (35.5) | 15,809 (28.8) | 10,229 (55.1) |
| **Male sex, N (%)** | 47,756 (65.1) | 35,781 (65.2) | 11,975 (64.5) |
| **Rural, N (%)** | 7,468 (10.2) | 5,010 (9.1) | 2,458 (13.2) |
| **Neighborhood income quintile, N (%)** |  |  |  |
| Low (q. 1-2) | 41,523 (56.6) | 30,362 (55.4) | 11,161 (60.1) |
| Medium (q. 3) | 12,510 (17.0) | 9,497 (17.3) | 3,013 (16.2) |
| High (q. 4-5) | 17,969 (24.5) | 13,930 (25.4) | 4,039 (21.8) |
| **Residential instability quintile, N (%)** |  |  |  |
| Low (q. 1-2) | 16,904 (23.0) | 13,169 (24.0) | 3,735 (20.1) |
| Medium (q. 3) | 11,240 (15.3) | 8,487 (15.5) | 2,753 (14.8) |
| High (q. 4-5) | 42,150 (57.4) | 31,059 (56.6) | 11,091 (59.8) |
| **Material deprivation quintile, N (%)** |  |  |  |
| Low (q. 1-2) | 17,680 (24.1) | 13,548 (24.7) | 4,132 (22.3) |
| Medium (q. 3) | 12,064 (16.4) | 9,161 (16.7) | 2,903 (15.6) |
| High (q. 4-5) | 40,550 (55.2) | 30,006 (54.7) | 10,544 (56.8) |
| **Ethnic concentration quintile, N (%)** |  |  |  |
| Low (q. 1-2) | 25,205 (34.3) | 17,864 (32.6) | 7,341 (39.6) |
| Medium (q. 3) | 12,942 (17.6) | 9,461 (17.2) | 3,481 (18.8) |
| High (q. 4-5) | 32,147 (43.8) | 25,390 (46.3) | 6,757 (36.4) |
| **Immigrant, N (%)** | 8,843 (12.0) | 7,210 (13.1) | 1,633 (8.8) |
| **Substance use disorder (ever), N (%)** | 36,378 (49.6) | 25,221 (46.0) | 11,157 (60.1) |
| **HIV positivity, N (%)** | 900 (1.2) | 760 (1.4) | 140 (0.8) |
| **HBsAG positivity ^a^, N (%)** | 463 (0.6) | 358 (0.7) | 105 (0.6) |
| **Aggregated diagnosis group categories, N (%)** |  |  |  |
| 0-3 ADG | 26,106 (35.6) | 19,157 (34.9) | 6,949 (37.4) |
| 4-7 ADG | 29,345 (40.0) | 22,351 (40.7) | 6,994 (37.7) |
| 8-10 ADG | 11,462 (15.6) | 8,643 (15.8) | 2,819 (15.2) |
| >11 ADG | 6,410 (8.7) | 4,648 (8.5) | 1,762 (9.5) |
| **Liver disease at the time of diagnosis,** **N (%)** |  |  |  |
| No cirrhosis | 57,568 (78.4) | 41,590 (75.8) | 15,978 (86.1) |
| Compensated cirrhosis | 5,058 (6.9) | 4,051 (7.4) | 1,007 (5.4) |
| Decompensated cirrhosis | 7,533 (10.3) | 6,477 (11.8) | 1,056 (5.7) |
| Hepatocellular carcinoma | 3,252 (4.4) | 2,736 (5.0) | 516 (2.8) |
| **Liver transplant,** **N (%)** | 994 (1.4) | 782 (1.4) | 212 (1.1) |
| **HCV genotype, N (%)** |  |  |  |
| Genotype-1 | 43,268 (58.9) | 32,780 (59.8) | 10,488 (56.5) |
| Genotype-2 | 7,391 (10.1) | 5,958 (10.9) | 1,433 (7.7) |
| Genotype-3 | 13,850 (18.9) | 9,576 (17.5) | 4,274 (23.0) |
| Genotype-4 | 1,384 (1.9) | 1,132 (2.1) | 252 (1.4) |
| Other/mixed | 1,534 (2.1) | 900 (1.6) | 634 (3.4) |
| **Treated, N (%)** | 34,932 (47.6) | 19,047 (34.7) | 7,571 (40.8) |
| **SVR, N (%)** | 28,110 (80.5) | 14,732 (77.3) | 6,269 (82.8) |

**- line 162: Why only 47 % received HCV treatment?**

*Response:* The cohort was identified between January 1, 1999, and December 31, 2018, and followed until May 31, 2021. Individuals were classified as having received treatment as per definition provided in the *Data sources* section:

*Data on HCV antiviral dispensation were sourced from Ontario Drug Benefit (ODB) using drug identification numbers (DIN) listed in* ***Table S1****. For those with a record of HCV antiviral drug dispensation from public drug plans, SVR was defined as a negative HCV RNA test result at least 12 weeks after the dispensation of the last HCV antiviral drug; those without a record of HCV antiviral drug dispensation from public sources but who had a final negative HCV RNA test result that took place after a positive HCV RNA were presumed to have achieved SVR through access to treatment via private insurance schemes external to ODB*.

We acknowledge that the treatment rates appear low, however, they are consistent with findings from other Canadian studies using administrative data [16, 17].

**- line 385+404: Do you mean liver-related death?**

*Response:* Thank you for the clarification. Yes, we were referring to liver-related death, and the wording has been revised accordingly throughout the manuscript.

**- line 402: Dot missing at the end of the sentence.**

*Response:* Thank you. A period has been added.

**REVIEWER #2:**

**The article presents a very interesting work, with a high number of patients about the impact of SVR obtained in the reduction of liver-death, but I have some issues that must be addressed:**

*Response:* Thank you for your kind appreciation of our work.

**1) The authors must describe very well in which manner they maintain the follow-up. Was only a clinical examination, or they performed an ultrasound, maybe an evaluation of liver fibrosis and steatosis after SVR was obtained?**

*Response:* We appreciate the reviewer’s comment. This study was a retrospective analysis using linked, population-based health administrative data from Ontario, Canada. As such, we did not conduct any direct clinical follow-up (e.g., clinical examination, ultrasound or any other diagnostic assessments) ourselves. Instead, this was a retrospective-prospective cohort design, where individuals were followed forward in time from the point of HCV viremia identification using routinely collected health records. The province’s publicly funded healthcare system ensures near-complete data coverage of the population, supporting the comprehensiveness and reliability of the analysis.

Clinical conditions such as cirrhosis, decompensated cirrhosis and advanced liver disease were identified using validated diagnostic codes available within these administrative datasets. The specific codes and definitions used are provided in the supplementary material (**Tables S2-S4**). These codes are derived from hospital discharge abstracts, physician billing claims, and other administrative sources that reflect encounters with the healthcare system. This methodology is described in the *Data Sources* section.

**2) The authors must explain if there is an impact of association of liver steatosis after SVR was obtained.**

*Response:* We thank the reviewer for this observation. However, assessing the impact or association of liver steatosis after SVR was not within the scope of our study. Our primary objective was to evaluate the association between SVR and liver-related mortality among individuals with hepatitis C, with a focus on key subgroups such as those with advanced liver disease (HCC, decompensated cirrhosis or liver transplant) and substance use.

**3) Liver steatosis was found to be an important factor that can cause advanced liver disease after SVR, due to the changes of behaviour in patients (more food-intake, appearances of metabolic syndrome and T2DM) which can cause liver-related events and death in time. How was the impact of liver steatosis in those subjects, after SVR was obtained?**

*Response:* We appreciate the reviewer’s insightful comment. As noted earlier, we did not specifically capture liver steatosis in our study. However, if individuals developed advanced liver disease following SVR, whether potentially due to steatosis, metabolic syndrome, or other causes, these events would have been captured using the diagnostic codes for advanced liver disease included in our analysis. However, we are unable to determine whether the advanced liver disease observed post-SVR was attributable to steatosis or to other causes.

To address the reviewer comment we have now added that in the limitation section.

*”Additionally, while cirrhosis and DC cases identified using validated algorithms, the accuracy of these definitions depend on the sensitivity and specificity of the algorithm, and cases of cirrhosis may be underestimated. We also did not account for liver steatosis, a condition that may lead to advance liver disease after the SVR, therefore we could not determine whether advanced liver disease was attributable to steatosis or other causes”*

**References**

1.	Erman A, Krahn MD, Hansen T, Wong J, Bielecki JM, Feld JJ, et al. Estimation of fibrosis progression rates for chronic hepatitis C: a systematic review and meta-analysis update. BMJ Open. 2019;9(11):e027491.

2.	Erman A, Sathya A, Nam A, Bielecki JM, Feld JJ, Thein HH, et al. Estimating chronic hepatitis C prognosis using transient elastography-based liver stiffness: A systematic review and meta-analysis. J Viral Hepat. 2018;25(5):502-13.

3.	Brown RS. Hepatitis C and liver transplantation. Nature. 2005;436(7053):973-8.

4.	Dore GJ, Feld JJ. Hepatitis C virus therapeutic development: in pursuit of "perfectovir". Clin Infect Dis. 2015;60(12):1829-36.

5.	van der Meer AJ, Veldt BJ, Feld JJ, Wedemeyer H, Dufour JF, Lammert F, et al. Association between sustained virological response and all-cause mortality among patients with chronic hepatitis C and advanced hepatic fibrosis. JAMA. 2012;308(24):2584-93.

6.	Biondi MJ, Feld JJ. Hepatitis C models of care: approaches to elimination. Can Liver J. 2020;3(2):165-76.

7.	Gambato M, Lens S, Navasa M, Forns X. Treatment options in patients with decompensated cirrhosis, pre- and post-transplantation. J Hepatol. 2014;61(1 Suppl):S120-31.

8.	Kwo PY, Mantry PS, Coakley E, Te HS, Vargas HE, Brown R, Jr., et al. An interferon-free antiviral regimen for HCV after liver transplantation. N Engl J Med. 2014;371(25):2375-82.

9.	Arain A, Robaeys G. Eligibility of persons who inject drugs for treatment of hepatitis C virus infection. World J Gastroenterol. 2014;20(36):12722-33.

10.	Grebely J, Dalgard O, Conway B, Cunningham EB, Bruggmann P, Hajarizadeh B, et al. Sofosbuvir and velpatasvir for hepatitis C virus infection in people with recent injection drug use (SIMPLIFY): an open-label, single-arm, phase 4, multicentre trial. Lancet Gastroenterol Hepatol. 2018;3(3):153-61.

11.	Grebely J, Hajarizadeh B, Dore GJ. Direct-acting antiviral agents for HCV infection affecting people who inject drugs. Nat Rev Gastroenterol Hepatol. 2017;14(11):641-51.

12.	Curry MP, O'Leary JG, Bzowej N, Muir AJ, Korenblat KM, Fenkel JM, et al. Sofosbuvir and Velpatasvir for HCV in Patients with Decompensated Cirrhosis. N Engl J Med. 2015;373(27):2618-28.

13.	Price JC, Terrault NA. Treatment of hepatitis C in liver transplant patients: interferon out, direct antiviral combos in. Liver Transpl. 2015;21(4):423-34.

14.	Pol S, Parlati L. Treatment of hepatitis C: the use of the new pangenotypic direct-acting antivirals in "special populations". Liver Int. 2018;38 Suppl 1:28-33.

15.	World Health Organization. Combating Hepatitis B and C to Reach Elimination by 2030. World Health Organization; 2016. <https://www.who.int/hepatitis/publications/hep-elimination-by-2030-brief/en/>. Accessed February 17, 2023.

16.	Erman A, Everett K, Wong WWL, Forouzannia F, Greenaway C, Janjua N, et al. Engagement with the HCV care cascade among high-risk groups: A population-based study. Hepatol Commun. 2023;7(9).

17.	Bartlett SR, Yu A, Chapinal N, Rossi C, Butt Z, Wong S, et al. The population level care cascade for hepatitis C in British Columbia, Canada as of 2018: Impact of direct acting antivirals. Liver Int. 2019;39(12):2261-72.
