# Supplementary material for: Effect of sustained virologic response on liver-related mortality among individuals living with hepatitis C by treatment era: A population-based retrospective cohort study
Source: PLoS One. 2025 Oct 6;20(10):e0333584. doi: 10.1371/journal.pone.0333584 (PMC12500089; doi:10.1371/journal.pone.0333584)
Supplement: Attachment — Submitted filename: Response_to_Reviewers_auresp_2.docx [file pone.0333584.s010.docx]

Dear Editor

Thank you for the opportunity to re-submit our revised manuscript, “Effect of sustained viral clearance on liver-related mortality among individuals living with hepatitis C by treatment era: a population-based retrospective cohort study” (**PONE-D-25-05248**) for publication in the *PLOS One.*

We thank the Reviewers for their further comments and proposed edits.

Please see below our detailed response to each of the comments. The changes are tracked in the revised manuscript.

Please kindly let us know if there are any further suggestions or comments.

Sincerely,

Beate Sander

Senior Scientist

University Health Network

200 Elizabeth Street, Toronto, ON M5G 2C4

E-mail: [beate.sander@uhn.ca](mailto:beate.sander@uhn.ca)

**Comments to the Author**

**REVIEWER #1:** The authors improved the readability of abstract and introduction and corrected mistakes. The authors implied most of the recommendations but forgot to correct SVR in the supplementary materials and I found some more flaws in the text.

**Response:** Thank you for your careful review and valuable recommendations during the review. We appreciate your acknowledgment of the improvements to the manuscript and your attention to the inaccuracies, which have now been corrected.

These mistakes or inaccuracies I suggest correcting:
Please stick to HCV RNA positivity, it is a more proper term than RNA positivity (line 175, 185...).

**Response: Thank you. This has now been corrected**

Explain N in bellow the tables (it was deleted in some table descriptions (eg. table 2,3,4) but I think it should stay there.

**Response: Thank you. This abbreviations has now been added.**

Throughout some tables HBsAG is written but both EASL and AASLD guidelines use abbreviation HBsAg (not capital G).

**Response: Thank you. This abbreviations has now been corrected.**

Throughout some tables abbreviation yrs. is used but not explained. Either explain it in the description of the table or write years as in the table 1.

**Response: Thank you. This abbreviation has now been spelled out in the tables.**

Throughout some tables abbreviation ref. is used but not explained in the description of the tables.

**Response: Thank you. This abbreviation has now been explained.**

Line 46: delete one bracket in )) = )

**Response: Thank you. This extra bracket has now been deleted.**

Line 104: formerly known or currently known as the Institute for Clinical Evaluative Sciences?

**Response: The current name is ICES. In 2018, the ICES formally adopted the initialism “ICES” as its official name. Before 2018, it was known as the Institute for Clinical Evaluative Sciences (ICES).**

Line 201: DAA era should be DAA-era if you want to stick to the terminology with dash which is used further in the text.

**Response: Thank you for your comment. We intended to use the spelling without the hyphen** (**DAA era), when "era" is a noun and with the hyphen, when era is an adjective (e.g., DAA-era cohort), and have checked and corrected throughout the text.**

Line 205: vs - dot is missing - vs.

**Response: Thank you we have added a dot, wherever applicable**

Line 233: space is missing after vs.

**Response: Thank you. This has been corrected.**

Line 243: dash is missing in pre-DAA-era.

**Response: Thank you for your comment. We intended to use the spelling without the hyphen** (**DAA era), when "era" is a noun and with the hyphen, when era is an adjective (e.g. DAA-era cohort), and have checked and corrected throughout the text.**

Line 276: CI: instead of C,I

**Response: Thank you. This has been corrected.**

Line 284+312: Time-to-liver-related mortality is not clear. There are too many dashes and I'm not sure if I understand the meaning of this term.

**Response: Thank you. For clarity we have revised the wording as “predictors of liver-related mortality”.**

Line 309: Do you mean higher incidence of liver-related death?.

**Response:** **Thank you, yes. This has been clarified in the text.**

Line 372: other word than realise would be more suitable

**Response:** **Thank you for the suggestion. We have replaced the word “realise” with “experience”.**

Line 393: What is the meaning of PHO HCV testing?

**Response: All data related to laboratory testing were collected from the Public Health Ontario (PHO) laboratory dataset. The PHO abbreviation was spelled out.**

Line 414: liver-related death.

**Response: Thank you. We have edited “liver-death” to “liver-related death”**

Line 426: correct liver-realted death to liver-related death.

**Response: Thank you we have corrected the spelling.**

Supplementary materials:

1. SVR is not corrected in the description of the abbreviations, it states sustained viral clearance.

**Response: Thank you we have corrected the term in the supplement.**

2. All tables are named S1-S7 but bellow the tables there is written table A1-A7 instead of S1-S7. In the main text all the tables are named only above the table but bellow the tables there is only the description but not Table xxx and the description. Please unify this and correct.

**Response: Thank you. This has been corrected for consistency in the supplementary materials and main text.**

Table S7: Sudy Cohort means study cohort?

**Response: Thank you, the typo has been corrected.**

**REVIEWER #2:** The article has been improved with the recommendations that I made for the first manuscript, and now can be accepted for publication.

**Response: Thank you for your positive feedback and for your helpful recommendations during the review process.**
